# Cinnamtannin B-1 Regulates Cell Proliferation of Spinal Cord Astrocytes and Protects the Cell from Oxygen-Glucose-Serum Deprivation/Reoxygenation-Induced Apoptosis

**DOI:** 10.3390/ijms140815827

**Published:** 2013-07-30

**Authors:** Zhiyong Chi, Xueling Ma, Guofeng Cui, Mingchao Li, Fuchun Li

**Affiliations:** 1 Department of Orthopaedics, the First Affiliated Hospital of Harbin Medical University, NO.23 Youzheng St., Nangang District, Harbin 150001, China; E-Mails: zhiyongchi@yeah.net (Z.C.); guofengcui@163.com (G.C.); mingchaoli@163.com (M.L.); 2 Department of Neurology, the Fourth Affiliated Hospital of Harbin Medical University, NO.37 Yiyuan Road, Nangang District, Harbin 150001, China; E-Mail: xuelingma103@yeah.net

**Keywords:** astrocytes, cinnamtannin B-1, ischemia/reperfusion, apoptosis

## Abstract

Astrocytes are important for protecting neurons in the central nervous system. It has been reported that some antioxidants could protect astrocytes from ischemia/reperfusion-induced dysfunction. Cinnamtannin B-1 is a naturally occurring A-type proanthocyanidin that exhibits anti-oxidant properties. In this study, we investigated the effects of cinnamtannin B-1 on spinal cord astrocytes. Astrocytes were subjected to oxygen-glucose-serum deprivation for eight hours followed by reoxygenation with or without cinnamtannin B-1. We found that cinnamtannin B-1 protected astrocytes from oxygen-glucose-serum deprivation and reoxygenation-induced apoptosis. Concurrently, cinnamtannin B-1 promoted the proliferation of astrocytes whereas the extracellular regulated protein kinase (ERK) inhibitor reversed this effect. The results indicated that cinnamtannin B-1 protects astrocytes from oxygen-glucose-serum deprivation/reoxygenation-induced apoptosis by promoting astrocyte proliferation via an ERK pathway. Therefore, as an anti-oxidant, cinnamtannin B-1 might provide extra benefit for astrocyte protection during ischemia/reperfusion in the central nervous system.

## 1. Introduction

Spinal cord ischemia is known to be one of the major causes of serious diseases and complications in the central nervous system (CNS). In some spinal cord ischemia animal models, catastrophic dysfunction and disability such as transient motor weakness or permanent paraplegia occurs [1–3]. However, the mechanism of ischemia and ensuing reperfusion-induced apoptosis of spinal neural cells remains unclear.

Astrocytes are one type of glial cell in the mammalian CNS, and the most numerous cell of the brain. Recent studies have shown a protective role for astrocytes in spinal cord injury [4,5]. Astrocytes could protect neurons in metabolic support, transmitter uptake and some other stresses. Thus, astrocyte dysfunction critically involves neuronal apoptosis in the process of ischemia/reperfusion [5–7]. In the process of ischemia/reperfusion injury, mitochondria oxidative stress has been identified as a contributor to disease and cell death [8]. As the most active cells in the CNS, astrocytes are particularly involved in this process.

**Figure 1:**
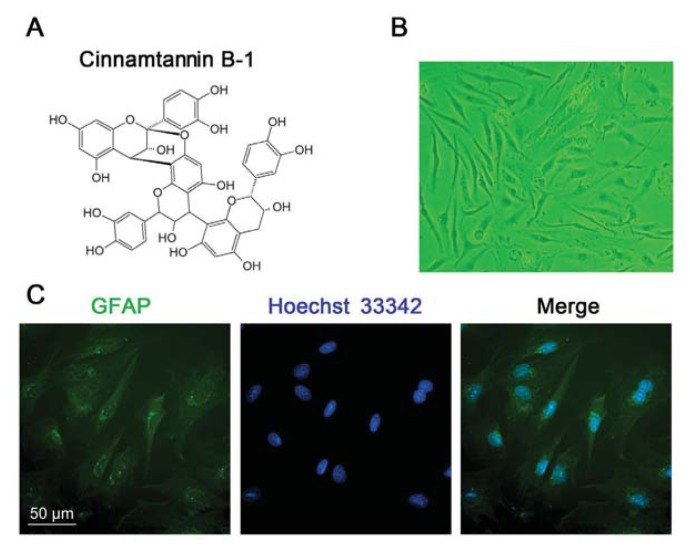
(**A**) The structure of cinnamtannin B-1; (**B**) Primary spinal cord astrocyte culture at 14 days; (**C**) Glial fibrillary acid protein (GFAP) staining. Over 95% of cells were stained with GFAP antibody (green fluorescence) and Hoechst 33342 (blue fluorescence). Scale bar = 50 μm and refers to all panels.

Some nonenzymatic antioxidants, such as natural therapies, arrest interest in the treatment of illnesses. Cinnamon is a spice currently being investigated as a potent antioxidant, which could provide benefit in disease like metabolic syndrome and type 2 diabetes [9,10]. Proanthocyanidins are widely available in cinnamon, which have been identified as naturally occurring flavonoids [11]. Also cinnamtannin B-1 is an A-type proanthocyanidin initially isolated from the bark of cinnamon, which is comprised of three monomeric units that form double links through C2→*O*→7 ether and carbon bonds (Figure 1A). Recently reports showed that cinnamtannin B-1 could exhibit strong antioxidant activity both *in vivo* and *in vitro* [12,13]. Cinnamtannin B-1 could generate potent antioxidant properties and contribute to the positive effect of cinnamon in type 2 diabetes. However, it has not been shown whether cinnamtannin B-1 could have an effect on the CNS in the ischemia/reperfusion injury.

In this study, we investigate the effect of cinnamtannin B-1 on primary cultured rat spinal cord astrocytes during oxygen-glucose-serum (OGSD) deprivation/reoxygenation-induced dysfunction. We also report that cinnamtannin B-1 can protect astrocytes from OGSD-induced apoptosis through the regulation of cell proliferation via a mitogen-activated protein kinase (MAPK) pathway.

## 2. Results and Discussion

### 2.1. Results

#### 2.1.1. Primary Culture of Rat Spinal Cord Astrocytes

Glial fibrillary acid protein (GFAP) is specially expressed in the astrocytes of the CNS [14]. Therefore, to identify the purity of the primary cultured spin cord astrocytes, immunocytochemistry was performed to show the GFAP-positive astrocytes in total primary cultured cells. After 14 days culture, over 95% of cells were stained positively for GFAP (Figure 1B,C).

#### 2.1.2. Cinnamtannin B-1 Protects Astrocytes from OGSD/Reoxygenation-Induced Apoptosis through ERK/Bcl-2 Pathway

**Figure 2:**
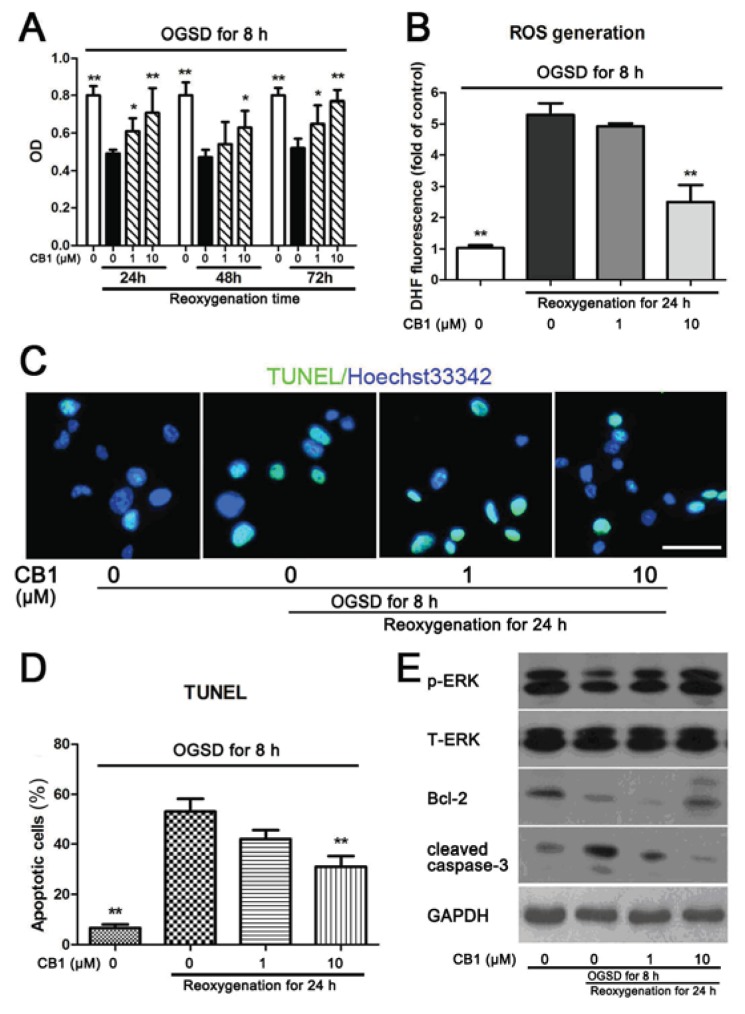
(**A**) Effect of cinnamtannin B-1 (CB1) on the viability of astrocytes. After 14 days culture, the astrocytes were subjected to oxygen-glucose-serum deprivation (OGSD) for 8 h, followed with/without reperfusion for 24, 48, 72 h in the absence or presence of 1 and 10 μM cinnamtannin B-1. After treatment, MTT assay was performed and the optical density (OD) was measured at a wavelength of 490 nm. *p < 0.05 and **p < 0.01 compared to vehicle-treated OGSD/reoxygenation group, respectively, *n* = 6; (**B**) Effect of CB1 on reactive oxygen species (ROS) generation. ROS level in each group was determined after OGSD 8 h following with/without reperfusion for 24 h. The fluorescence of dichlorofluorescein (DCF) was measured. *p < 0.05 and **p < 0.01 compared to vehicle-treated OGSD/reoxygenation group, *n* = 3; (**C**) Terminal deoxynucleotidyl transferase dUTP nick end labeling (TUNEL) staining. TUNEL positive cells were marked with green fluorescence, and the nuclei of cells were stained blue by Hoechst 33342. The merged figures were shown and the scale bar = 50 μm and referred to all panels; (**D**) The statistical analysis of TUNEL positive cells. **p < 0.01 compared to vehicle-treated OGSD/reoxygenation group, *n* = 6; (**E**) Immunoblot analysis. Following OGSD for 8 h ensuing with/without reperfusion treatment for 24 h, the cell lysate from four groups were collected and immunoblot analysis of phosphorylation extracellular regulated protein kinase (p-ERK), total ERK (T-ERK), Bcl-2, cleaved caspase-3 and GAPDH were performed. Three independent experiments were performed and a representative experiment was depicted.

It has been reported that OGSD/reoxygenation could inhibit the viability of astrocytes [15]. A 3-(4,5-dimethylthiazol-2-yl)-2,5-diphenyltetrazolium bromide (MTT) assay was performed to investigate the effect of cinnamtannin B-1 on the viability of astrocytes after OGSD/reoxygenation for different terms. We found that after 8 h OGSD followed by reoxygenation, the viability of astrocytes decreased significantly compared to control cells. However, there was no obvious difference among the cells reoxygenated for 24, 48, 72 h (Figure 2A). In contrast, the viability of cinnamtannin B-1 (10 μM) treated cells was significantly higher than the vehicle-treated group after OGSD/reoxygenation (Figure 2A). In addition, treatment with different concentrations of cinnamtannin B-1 had no effect on apoptosis or viability of astrocytes (data not shown).

In the process of OGSD/reoxygenation, increased production of reactive oxygen species (ROS) is found to cause oxidative stress and cell death [16]. Therefore, the ROS generation under OGSD for 8 h/reoxygenation for 24 h was measured. OGSD/reoxygenation obviously increased the intracellular level of ROS (Figure 2B). However, in the presence of 10 μM cinnamtannin B-1, the ROS generation in OGSD/reoxygenation treated cells was inhibited significantly compared to the OGSD/reoxygenation treated only group, which suggested that cinnamtannin B-1 reduced the ROS generation under ischemia/reperfusion.

Since cinnamtannin B-1 protected astrocytes from oxygen-glucose deprivation and reduced the ROS generation in astrocytes, we investigated the apoptotic ratio of cells under OGSD for 8 h and reoxygenation for 24 h in the presence or absence of cinnamtannin B-1 using Terminal deoxynucleotidyl transferase dUTP nick end labeling (TUNEL) staining. Although no obvious change was found in the 1 μM cinnamtannin B-1 treated group compared to the OGSD/reoxygenation treated only group, the compound reduced the number of apoptotic cells under OGSD/reoxygenation at a concentration of 10 μM (Figure 2C).

In the process of cell apoptosis, especially in the ischemia/reperfusion-induced cell death, mitochondria oxidative stress plays an important role [16,17]. To identify whether cinnamtannin B-1 protected astrocytes from OGSD/reoxygenation by reducing mitochondria apoptosis, the expression levels of some proteins involved in mitochondria oxidative stress-induced apoptosis were analyzed. As shown in Figure 2D, after the cells were treated for OGSD 8 h/reoxygenation 24 h, the expression level of p-ERK, Bcl-2 was reduced. Moreover, the expression level of cleaved caspase-3 was found to increase. We found that cinnamtannin B-1 treated cells had an elevated expression level of p-ERK and Bcl-2 in comparison with the OGSD/reoxygenation-treated only group. Also, the activation of caspase-3 was inhibited by cinnamtannin B-1 in a dose-dependent manner. Taken together, cinnamtannin B-1 protected primary cultured astrocytes from OGSD/reoxygenation-induced apoptosis probably by increasing the ERK phosphorylation and Bcl-2 expression level.

#### 2.1.3. Cinnamtannin B-1 Regulates the Proliferation of Spinal Cord Astrocytes through an ERK Pathway

**Figure 3:**
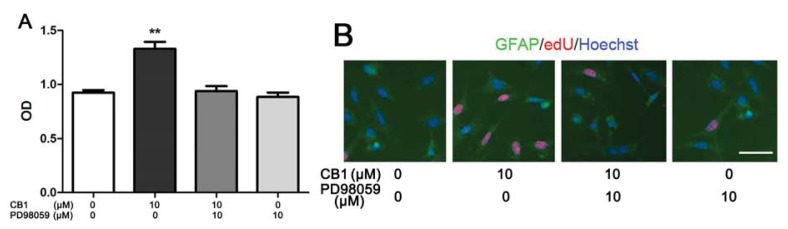
(**A**) Effect of cinnamtanin B-1 (CB1) on the proliferation of astrocytes. The primary cultured astrocytes were incubated with 10 μM cinnamtanin B-1 or 10 μM PD98059 or co-incubated with 10 μM cinnamtanin B-1 and 10 μM PD98059 for 24 h, then the MTT assay was performed. **p < 0.01 compared to control group, *n* = 6; (**B**) EdU staining. EdU staining was performed after cells were treated as above. The EdU positive cells were stained with red fluorescence, the GFAP was marked with green fluorescence, the nuclei of cells were stained with blue fluorescence. Scale bar = 50 μm and refers to all panels.

The level of ERK phosphorylation was found elevated in the cinnamtannin B-1 treated cells under OGSD/reoxygenation conditions. ERK phosphorylation plays a key role in the MAPK pathway, which is associated with cell proliferation [18]. Thus, we detected the probable effect of cinnamtannin B-1 on the proliferation of astrocytes. As depicted in Figure 3, it was found that cinnamtannin B-1 could significantly improve the cell proliferation of astrocytes at a concentration of 10 μM. Also, the MAPK inhibitor, PD98059, reversed this effect (Figure 3A). In addition, the proliferation rate of astrocytes was determined by an EdU kit. In the EdU assay, the proliferating nuclei could be marked with fluorescence probes [19]. In accordance with the result of the MTT assay, it was found that 10 μM cinnamtannin B-1 stimulated the proliferation of astrocytes. However, PD98059 inhibited this effect (Figure 3B). To sum up, cinnamtannin B-1 regulated the proliferation of spinal cord astrocytes at least partially through ERK activation.

### 2.2. Discussion

Proanthocyanidins, as principal components in cinnamon, are primarily known for their antioxidant activity. [20]. Cinnamtannin B-1 is a naturally occurring proanthocyanidin isolated from cinnamon and some other plants [21]. As a useful antioxidant, cinnamtannin B-1 has three flavan-3-ol monomeric units, and its protective effects against ROS have been reported in many types of cells [21–24]. It has been reported that cinnamtannin B-1 possesses potent antioxidant properties in type 2 diabetes and mediates some beneficial effects [25]. However, the possible effect of cinnamtannin B-1 in ischemia/reperfusion-induced apoptosis has not been studied. In this study, we found that cinnamtannin B-1 could protect primary cultured astrocytes from OGSD/reoxygenation-induced injury.

Oxidative stress plays a key role in the process of cell death and is thought to be involved in the development of numerous diseases, such as ischemia/reperfusion injury and some neurodegenerative diseases [8,26]. In this process, the generation of ROS induces cell injury. The major intracellular ROS produced by mitochondria inhibits components of the respiratory chain [27], promoting the release of cytochrome c and activating the apoptosis-inducing factor (AIF) [28,29]. Increased intracellular ROS and ROS-mediated cell apoptosis in the spinal cord has been reported [30]. We also found that the generation of ROS in OGSD/reoxygenation-treated primary cultured astrocytes is extremely higher than in control cells, and the antioxidant, cinnamtannin B-1 reduced the increased level of ROS in OGSD/reoxygenation-treated cells, which can be considered as a factor in the protective effect of cinnamtannin B1 in cell survival.

The MAPK pathway has been found to be critically related to the process of cell proliferation and apoptosis [31,32]. ERK activation is involved in astrocytes associated with ROS generation [33]. However, imbalances in ROS production could result in serious radical-induced damage. Furthermore, numerous pro-apoptotic and anti-apoptotic proteins on the surface of the mitochondrial membrane are associated with this process. Radical-induced cytochrome c could activate caspase-3 to generate cell death [34]. Meanwhile, an anti-apoptotic protein Bcl-2 could prevent cell apoptosis possibly through the suppression of oxyradical-induced membrane injury [35,36]. We determined the apoptosis rate of OGSD/reoxygenation-treated cells in the presence/absence of cinnamtannin B-1, and found that cinnamtannin B-1 could protects astrocytes from OGSD/reoxygenation-induced apoptosis. Reduced ROS generation and restored ERK activation might be involved in the process, which leads to an improved Bcl-2 expression and a decreased caspase-3 activation level.

Astrocytes can support neuronal survival through regulation of ROS via activation of some endogenous antioxidant such as glutathione (GSH) [37,38]. Antioxidative function deficiency of astrocytes may lead to neuronal injury [39,40]. Some diseases, including spinalmuscularatrophy (SMA) and amyotrophic lateral sclerosis (ALS) were found to be associated with function loss of astrocytes in the spinal cord [41]. Thus, using exogenous antioxidants could be beneficial for the protection of the CNS.

In the process of OGSD/reoxygenation-induced apoptosis, we showed that cinnamtannin B-1 treated cells had an elevated phosphorylation level of ERK. Therefore, we investigated whether cinnamtannin B-1 has an effect on normal cultured astrocytes. Interestingly, 10 μM cinnamtannin B-1 increased cell viability remarkably compared to normal cultured astrocytes. PD98059, an ERK phosphorylation inhibitor reversed this effect. Furthermore, the EdU staining also suggested that cinnamtannin B-1 could promote the proliferation of astrocytes, PD98059 blocked this effect. To sum up, cinnamtannin B-1 had an effect on astrocytes via a MAPK pathway, at least through ERK phosphorylation.

## 3. Materials and Methods

### 3.1. Reagents

Cell culture reagents, Dulbecco’s modified Eagle’s medium (DMEM), trypsin, fetal bovine serum (FBS), penicillin and streptomycin were all purchased from GIBCO, USA. 3-(4,5-dimethylthiazol-2-yl)-2,5-diphenyltetrazolium bromide (MTT), PD98059, Hoechst33342 were purchased from Sigma-Aldrich, USA. Reactive oxygen species (ROS) detection kit was purchased from Beyotime Biotechnology, CN. Cinnamtannin B-1 (epicatechin-4β→8 2β→*O*→7)-epicatechin-(4α→8)-epicatechin) isolated from *Laurus nobilis* L. came from Alexis Corporation, Switzerland (Figure 1A). TUNEL staining kit came from Roche. The EdU staining kit and Alexa Fluor 488-conjugated goat anti-rabbit IgG antibody were purchased from Invitrogen, USA. GFAP antibody was purchased from Santa Cruz Biotechnology, GAPDH, phosphor-ERK, ERK, Bcl-2 and caspase-3 antibodies were from Cell signaling technology. Peroxidase-labeled goat anti-rabbit IgG was purchased from Jackson Immuno Research.

### 3.2. Cell Culture

Primary rat spinal cord astrocytes culture was performed as previously described [42]. Briefly, newborn (1 day after birth) Sprague-Dawley rats were used in this experiment. Spinal cords were carefully dissected with the meninges removed. Spinal cords were dissociated in trypsin (0.25%) for 5 min at 37 °C, then the suspension was centrifuged for 5 min at 1500 rpm. The supernatant was removed and the cells were filtered with a 200 μm mesh sieve. After that, cells were seeded on a 6-well plate in DMEM with 10% FBS, 100 IU/mL penicillin and 100 μg/mL streptomycin. The cells were cultured at conditions of 37 °C and 5% CO_2_. All experiments were performed after 14 days culture.

OGSD was performed as previously described [43]. Briefly, the culture media were removed and cells were washed with PBS before OGSD. Cells were exposed to 95% N_2_ and 5% CO_2_ in a hypoxia gas chamber (Russkin, Bridgend, UK) without serum and glucose in DMEM for 8 h. Then the plates were taken out from the chamber, the glucose and FBS were added and the cells were incubated under normal conditions for different times to generate reoxygenation.

### 3.3. MTT Assay and Determination of ROS Generation

Astrocytes were planted in 96-well plates (15,000 cells per well) and treated as above. The cell viability was determined by MTT assay. Following treatment, the MTT reagents (0.5 mg/mL) were added to each well over 4 h, then dimethyl sulfoxide (DMSO) was added to dissolve the insoluble purple formazan product. The absorbance at a certain wavelength, 490 nm, was measured by MK3 multiskan (Thermo Fisher, Shanghai, China). ROS generation in each group of treated cells was measured by ROS detection kit following its protocol. Briefly, 2′,7′-dichlorofluorescein diacetate (DCFH-DA) could be converted to fluorescent dichlorofluorescein (DCF) in the presence of ROS, the DCF fluorescence distribution of 15,000 cells per well was measured by Flexstation 3 (Molecular device, Sunnyvale, USA) at 488 nm of excitation wavelength and 535 nm emission wavelength.

### 3.4. Immunofluorescence

The primary cultured spinal cord astrocytes were stained with the astrocytic marker glial fibrillary acid protein (GFAP) before experiments. Cells were planted on glasses in 6-well plates at 500,000 per well and fixed overnight in 4% paraformaldehyde for immunofluorescence. The cells were washed with phosphate buffered saline (PBS) three times followed by blocking in 1% bovine serum albumin (BSA) for 15 min at room temperature. The monoclonal primary antibody rabbit anti-GFAP (dilution 1:100) was added on the glasses and incubated overnight at 4 °C. Following multiple PBS washes, the cells were incubated with Alexa Fluor 488-conjugated goat anti-rabbit IgG (dilution 1:400) in the dark for 2 h at room temperature. After PBS washes, the nuclei of cells were stained by Hoechst33342. In the EdU staining, the EdU kit was used following the protocol before the glasses were mounted. The images were captured by a microscope (DP70, Olympus, Tokyo, Japan).

### 3.5. TUNEL Staining

Cells were cultured on coverglasses in six-well plates. After treatment, the apoptotic cells were stained by TUNEL kit followed the protocol. The apoptotic cells were marked with green fluorescence, and all the cells were stained with Hoechst33342. The ratio of apoptotic was calculated as the apoptotic cells divided by the total cells.

### 3.6. Western Blot Analysis

After treatment, the cells were lysed by RIPA lysis buffer (Beyotime, Shanghai, China) according to the protocol. Western blot was carried out as described with minor modification [44]. The dilution of the primary antibodies was: phosphor-ERK (1:1000), ERK (1:1000), Bcl-2 (1:500), caspase-3 (1:500), GAPDH (1:5000), respectively.

### 3.7. Statistical Analysis

Data were expressed as the mean ± SD. One-Way ANOVA analysis was performed and a value of *p* < 0.05 was considered significant.

## 4. Conclusions

We found that cinnamtannin B-1 could protect astrocytes from OGSD/reoxygenation-induced apoptosis by regulation of ROS generation, and possibly promoted cell proliferation via a MAPK pathway. Also, these results suggested cinnamtannin B-1 could be beneficial in the treatment of some diseases associated with ischemia/reperfusion.
